# The Small Posterior Cranial Fossa Syndrome and Chiari Malformation Type 0

**DOI:** 10.3390/jcm11185472

**Published:** 2022-09-17

**Authors:** Enver I. Bogdanov, Aisylu T. Faizutdinova, John D. Heiss

**Affiliations:** 1 Department of Neurology and Rehabilitation, Kazan State Medical University, 420012 Kazan, Russia; 2 Surgical Neurology Branch, National Institute of Neurological Disorders and Stroke, National Institutes of Health, Bethesda, MD 20892, USA

**Keywords:** small posterior cranial fossa, Chiari malformation type 0, Chiari malformation type 1, cerebellar tonsil herniation, morphometric analysis, syringomyelia

## Abstract

Patients showing typical Chiari malformation type 1 (CM1) signs and symptoms frequently undergo cranial and cervical MRI. In some patients, MRI documents >5 mm of cerebellar tonsillar herniation (TH) and the diagnosis of CM1. Patients with 3–5 mm TH have “borderline” CM1. Patients with less than 3 mm of TH and an associated cervical syrinx are diagnosed with Chiari “zero” malformation (CM0). However, patients reporting CM1 symptoms are usually not diagnosed with CM if MRI shows less than 3–5 mm of TH and no syrinx. Recent MRI morphometric analysis of the posterior fossa and upper cervical spine detected anatomical abnormalities in and around the foramen magnum (FM) that explain these patients’ symptoms. The abnormalities include a reduced size of the posterior fossa, FM, and upper cervical spinal canal and extension of the cerebellar tonsils around the medulla rather than inferior to the foramen magnum, as in CM1. These morphometric findings lead some neurologists and neurosurgeons to diagnose CM0 in patients with typical CM1 signs and symptoms, with or without cervical syringes. This article reviews recent findings and controversies about CM0 diagnosis and updates current thinking about the clinical and radiological relationship between CM0, borderline CM1, and CM1.

## 1. Introduction

Hans Chiari described four different anomalies of the cerebellum that now carry his name. Subsequently, other investigators added subclassifications, and current classifications include Chiari I (subtypes Chiari 0, Chiari 0.5, Chiari 1.5), Chiari II, Chiari III (subtype Chiari 3.5), Chiari IV, and Chiari V malformations [1,2].

The definitions and systems of classification of the Chiari malformation type 1 (CM1) spectrum abnormalities are controversial and broadly discussed in contemporary literature [3,4,5,6,7,8,9,10]. Some researchers believe that CM0 is a unique posterior cranial fossa (PCF) malformation [2,3,11,12,13]. Others believe that Chiari malformation type 0 (CM0) is a subtype of CM1 and that CM0 subtyping is imprecise and lacks clinical relevance [9]. Recently, 38.9% of 63 international experts defined CM0 as “a volumetrically small posterior fossa, obliteration of the cisterna magna, cerebellar tonsils positioned at the foramen magnum, and the presence of a slit-like syringomyelia (SM) cavity within the cervical spinal cord” [4]. The inability to reach a consensus among all experts may reflect concerns about imprecise diagnostic criteria leading to incorrect CM0 diagnoses and unnecessary surgery [4]. In another study, 81.3% of 34 experts reached agreement on this CM0 definition: “obliteration of the cisterna magna (due to arachnoid adhesions) and/or volumetrically small posterior fossa, with cerebellar tonsils positioned at the foramen magnum and a syringomielia in the cervical spinal cord” [14]. The posterior fossa characteristics of CM0 mentioned above can be present without syringomyelia in patients with CM1-like clinical manifestations. Clinical and radiological features of two subtypes of CM0 with and without syringomyelia were described [15,16]. These subtypes of CM0 need further elaboration to differentiate them from the much larger group of people with minimal tonsillar ectopia and mild or absent symptoms.

This article aims to review findings and controversies about CM0 diagnosis. It will update current thinking about the clinical and radiological findings in symptomatic patients with cerebellar tonsillar ectopia, a small cranial posterior fossa, and CM0, borderline CM1, or CM1. Finally, it will contrast the posterior fossa morphology of patients with CM with that of asymptomatic people with cerebellar tonsillar ectopia.

## 2. Materials and Methods

**Figure 1:**
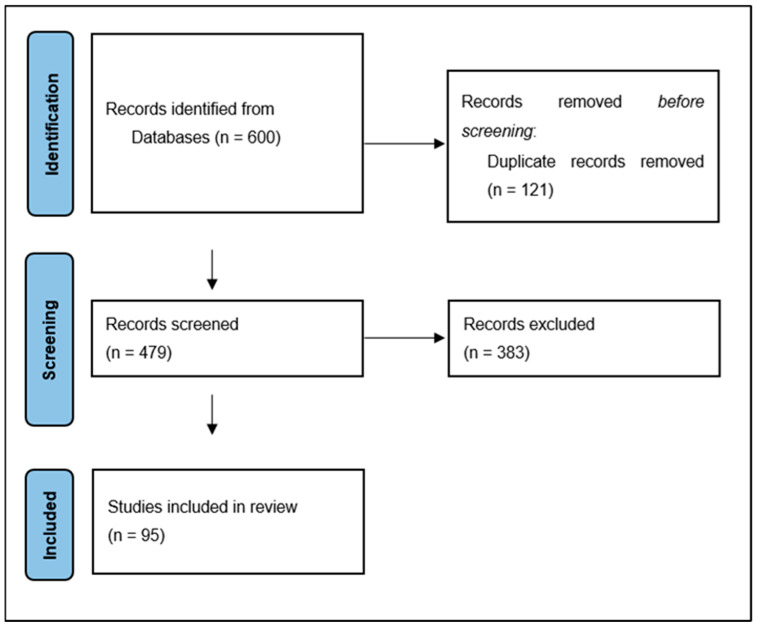
Flow diagram of the search strategy.

This literature review was conducted based on an electronic search of the MEDLINE database using PubMed (https://pubmed.ncbi.nlm.nih.gov) and Google Scholar (accessed on 1 February 2022). The following terms were used in the Electronic Search Strategy: “Chiari type zero malformation”, “small posterior fossa syndrome”, “tight cisterna magna”, “posterior cranial fossa crowdedness”, “borderline tonsillar herniation”, “(posterior fossa morphometry) OR (craniometry) AND (Chiari malformation)”, “low-lying cerebellar tonsils”, and “Chiari malformation classification”. The search included published articles through March 2022. A total of 600 sources were received. The subsequent screening included the removal of duplicate publications, publications published before 1980 (due to the low prevalence of MRI-verified observations), and links to inaccessible full-text sources. As a result, 95 publications were selected (Figure 1), including prospective (n = 7), retrospective (n = 25), cohort (n = 7) studies, family studies and genetic studies (n = 7), case-control studies (n = 27), and reviews and discussions (n = 16). Pertinent historical articles (n = 2) and book chapters (n = 4) provided additional information and perspective for the review.

## 3. Results

### 3.1. Symptomatic Conditions with Low-Lying Cerebellar Tonsils Found by the Article Search and Selection Process

Milhorat and colleagues studied radiographic PCF morphology in 741 patients with TH. In 52% of these patients, PCF size and volume were significantly smaller than normal. CM1 with a small PCF was named classical (primary) CM1 [17]. However, occipital bone size and PCF volume were normal in the other 48% of patients with cerebellar tonsillar herniation (TH), who had occipitoatlantoaxial joint instability (34%), tethered cord syndrome (7%), intracranial mass lesions (4%), and lumboperitoneal shunts (3%) [17]. The authors referred to these latter forms as secondary CM1 or CM1 mimics.

Several studies have reported patients can have CM1-like symptoms without having significant TH [15,16,18,19,20,21,22]. These patients shared many PCF morphologic traits of classical CM1, including isolated hypoplasia of occipital bone, a small PCF, and PCF constriction from a mismatch between the volume of the PCF and its contents [23,24,25,26]. Some authors use the CM0 or CM1-like terms for patients with CM1 symptoms, diminished PCF volume, and minimal tonsillar descent [20,21,22,27], whereas others use CM0 only for patients with minimal tonsillar ectopia and cervical syringomyelia [3].

### 3.2. Characteristics of Studies Differentiating CM0 from CM1 and Other Conditions

Early MRI studies reported that the lowest normal tonsillar position was 3 mm of TH, with a sensitivity of 96% and specificity of 99.5%. An MRI finding of ≥5 mm of TH was adopted as the diagnostic criteria for CM1 because it predicted CM1 symptoms with 100% sensitivity and 98.5% specificity [28]. Later investigations showed that the discrete 5 mm cutoff was less specific for CM1 symptoms than earlier reported [16,29]. Most but not all individuals with typical CM1 complaints have at least 5 mm of TH below the foramen magnum (FM), an amount of TH that lies in the tail of the normal distribution of asymptomatic persons [29]. Patients with MRI-diagnosed classic CM1 may exhibit symptoms of varying severity or be asymptomatic [30,31,32]. More recent studies report that a radiographic diagnosis of CM1 based on at least 5 mm of TH on midsagittal MRI is relatively insensitive and nonspecific for predicting CM1 symptoms [33,34]. CM1 clinical manifestations are associated with factors other than TH, including PCF shallowness, cerebrospinal fluid (CSF) space tightness, CSF pathway obstruction, brainstem and cerebellar compression, and syringomyelia [35].

Typical CM1-like clinical manifestations can develop in patients with TH less than 5 mm and a small posterior cranial fossa (SPCF) [15,16,19,21,22]. CM1-like symptoms, TH less than 5 mm, and hindbrain overcrowding occurred in 9% of patients in a CM1 surgical series and 33% in a neurology institutional database [18,24]. Patients with CM1-like symptoms and TH of 3–5 mm may be diagnosed with borderline CM1 on midsagittal MRI [36]. CM0 diagnosis initially required patients to have cervical syringomyelia with <3 mm of TH before and syrinx resolution after PCF decompression [2,3]. In CM0, many authors proposed that SPCF reducing the cross-sectional area around the FM was the factor responsible for symptom development [15,20,27,37,38]. Several studies examined the radiologic characteristics of CM0 [15,22,27,37]. In one study, in six 3–16 year old children with CM0 and syringomyelia (CM0-syr), the obex was located more than two standard deviations below its normal position [13]. Bogdanov et al. measured 11 parameters in 17 adult CM0-syr patients and demonstrated that patients with CM0-syr had SPCF and narrow CSF spaces such as patients with classical CM1 and syringomyelia (CM1-syr) [27]. A recently published definition of CM0 includes (1) obliteration of the cisterna magna, (2) volumetrically SPCF, (3) cerebellar tonsils positioned at the FM, and (4) cervical syringomyelia [4,14].

**Figure 2:**
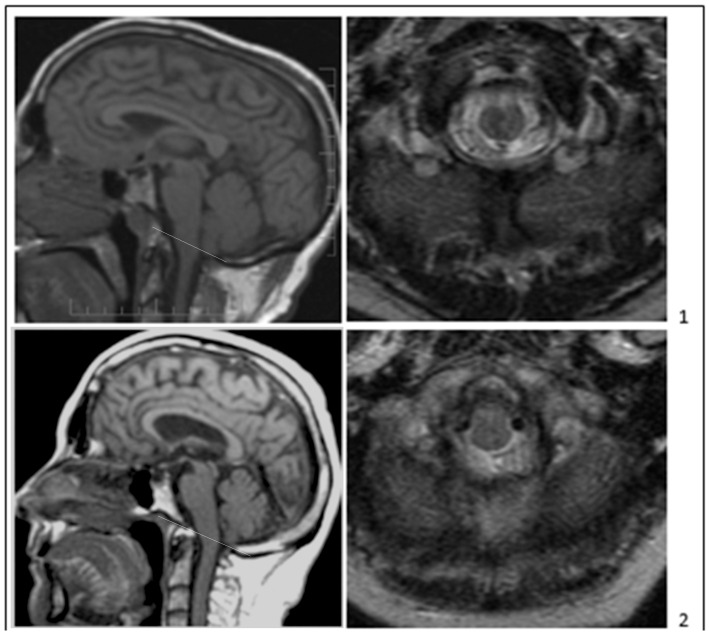

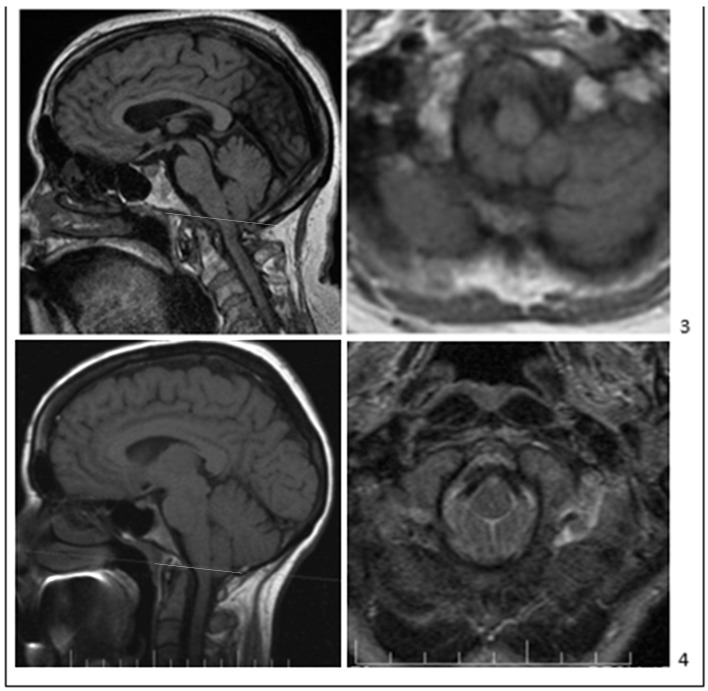
Types of CM0 without syringomyelia. The McRae line (white line) is shown on the T1-weighted sagittal images (left panels). Axial images at McRae’s line are shown (right panels) on T1-weighted (**3**) and T2-weighted (**1**,**2**,**4**) images. Adult symptomatic CM0-only patients with tonsillar herniation ≤2 mm (panels (**1**–**3**)) and borderline CM1-only patients with tonsillar herniation 3 mm (**4**). All patients demonstrate short bones (CL and SO < 40 mm), crowdedness of the PCF, tight foramen magnum, and CM1-like clinical manifestations: transient localized suboccipital cough-related headaches (**1**) or constant and transient suboccipital headaches (**2**–**4**), truncal ataxia, vertigo, dizziness, and upper motor neuron signs. The sagittal images in (**1**,**3**) (left panels) also show spinal canal narrowing at the level of the odontoid.

Some investigators have used CM0 to refer to patients with and without syringomyelia with minimal or absent TH, radiological features of SPCF, and typical clinical signs and symptoms of CM1, including occipital headache, posterior cervical pain, and cerebellomedullary dysfunction (Figure 2) [19,21,39,40]. Sekula et al. reported 22 patients with CM1-like signs and symptoms, an abnormally short clivus, abnormally wide angle of the tentorium to the Twining line, less than 3 mm of TH, and no syringomyelia [22]. Heffez et al. documented brain stem compression by radiologic and surgical criteria in 97 adult patients with Chiari-like symptoms who had TH within but not below the FM. Only 4% of those patients had syringomyelia [16]. These investigations support classifying CM0 without syringomyelia as a classical CM1 variant because CM0 and CM1 share findings of an SPCF and caudally located obex [3,5,13,15,27,41,42]. In addition, studies of monozygotic twins, triplets, and families affected by CM1 demonstrate other members affected with CM0, suggesting that the families have a heritable SPCF trait with variable TH expression [42,43,44]. TH variability among monozygotic triplets and twins suggests that the environment and epigenetics influence SPCF expression and the extent of TH. In families with CM1, the occipital hypoplasia phenotype documented by SPCF radiological traits was inherited more consistently than tonsillar descent or CM1 clinical signs and symptoms [38,42,45,46]. Families with abnormal PCF morphometric features had five patients with CM0 and syringomyelia and seven with CM1 [42]. In our study of seven families with multiple members affected by CM1 spectrum disorders, 46 subjects had MRI-morphometric findings of SPCF, 17 of which had CM1 (TH ≥ 5mm) with or without syringomyelia, 9 had borderline CM1 (TH 3–5 mm) with or without syringomyelia, and 20 had CM0 (TH < 3 mm) with or without syringomyelia [47]. Occipital hypoplasia reduces PCF volume without affecting neural content volume, resulting in neural crowding, herniation of the tonsils into or below the FM, and Chiari-like clinical manifestations with or without syringomyelia [5,15,27]. Occipital hypoplasia arises from insufficient growth of the para-axial mesoderm, underdevelopment of the occipital somites, and abnormal development of the basichondrocranium. Para-axial mesodermal derangement in CM1 and CM0 may extend to the cervical spine [48,49,50,51,52]. Steeper spinal canal tapering in CM0 and CM1 may create CSF pressure craniocaudal gradients encouraging syrinx formation or cervical paraspinal muscle alterations [15,48,49,53].

Most studies of CM0 and CM1 demonstrate abnormally small PCF bone length, PCF volume, and crowding of the PCF neural structures [15,17,20,22,24,25,27,41,54,55,56,57,58,59,60,61,62]. In contrast, other studies reported similar PCF volumes in CM1 and normal controls [8,63,64]. The discrepancies across CM1 studies of PCF linear, angular, cross-sectional area, and volume measurements could arise from the different study populations and the inclusion of patients with acquired TH. Patients with a normal-volume PCF can develop CM1 from obstruction of the FM and foramen Magendie by arachnoid veils and adhesions, creating a craniospinal pressure gradient [65].

Nishikawa et al. radiologically examined 500 symptomatic patients with CM1 with TH ≥ 5 mm below the FM, 50 patients having CM1-like symptoms with low-lying tonsils (<5 mm), and a healthy control group [20]. They identified three subtypes of CM1: (1) type A was characterized by a normal PCF volume, normal volume around the FM, and normal occipital bone size. This CM1 subtype was associated with hindbrain ptosis and not shallowness of the PCF. It arose from conditions such as tethered cord syndrome, craniovertebral junction instability, and increased intracranial pressure [66]; (2) type B was characterized by normal PCF overall volume, reduced PCF volume around the FM, and reduced PCF bone size; (3) type C was characterized by small PCF volume, reduced PCF volume around the FM, and small occipital bone size. Thus, the PCF was underdeveloped in the B and C CM1 subtypes. CM1 type A arose from secondary (acquired) TH unrelated to occipital hypoplasia. In the group with TH < 5 mm, named “CM-absence” by the authors, the PCF volume around the FM was reduced, but the entire PCF volume was normal. CM-absence, referred to as CM0 or borderline CM1 by other authors, also included narrowing of the FM CSF pathways and compression of the brain stem by ectopic tonsils and scarred arachnoid [16,39,40,41,67]. The CM-absence group shared the abnormal PCF morphology seen in the CM1 B and C subtypes, suggesting that CM-absence fits the classic CM1 disease spectrum.

In a study of 137 CM1 symptomatic patients (51% with syringomyelia) and 14 CM0 symptomatic patients (64% with syringomyelia), both CM1 and CM0 patients had an abnormally short clivus, reduced sagittal PCF area, and a narrowed (more acute) tentorium-occipital bone angle. The clivo-axial (Wackenheim’s) angle was significantly wider in the CM0 group compared to the CM1 group, but other PCF morphometric measurements were similar between CM0 and CM1 [39]. In a study of 7 symptomatic CM0 patients, 3 (43%) with syringomyelia, and 141 symptomatic CM1 patients, 77 (55%) with syringomyelia, the CM0 patient group had a longer clivus and supraoccipital bone than the CM1 group, but smaller PCF cross-sectional area and a shorter clivus than the control group [40]. Defining factors of CM0 in other studies were (1) cerebellar tonsils located within but not below the FM, (2) brainstem caudal displacement and compression, (3) cerebellar tonsils obstructing CSF flow, and (4) membranes, adhesions, and scarring around the FM [16,37,68,69]. Heffez et al. studied a series of patients with Chiari-like symptoms and divided them into three groups by their amount of TH: (1) from 0 to less than 3 mm, (2) 3–5 mm, and (3) >5 mm [16]. Only 4% of patients in Group 1 and 7.7% in the other two groups had syringes. These authors noted neural compression within the FM [16]. Group 1 patients had cerebellar tonsils within the FM compressing the brain stem and more severe symptoms than Groups 2 and 3. The presence of neurologic signs was not related to TH extent (mm) by logistic regression analysis. All groups received the same type of surgical decompression procedure for CM1. The surgeon noticed no difference in intraoperative anatomy and physiology, such as deformity of the brain stem, neuroma of the C1 root, neural compression, posterior inferior cerebellar artery compression, or change in brain stem auditory evoked potentials between groups. Surgical decompression benefited all groups similarly and without regard for TH [67]. The authors concluded that typical CM1 signs and symptoms are more critical than TH extent in diagnosing CM1 spectrum disorders and predicting surgical outcomes.

To better understand how anatomical features of CM0 result in CM1-like clinical manifestations, we conducted a morphometric study of adult symptomatic patients with CM1 and CM0 [15]. The clivus length and supraocciput length in all patients were ≤40 mm, one standard deviation below the control group means [15,19,57]. Patients were divided by MRI findings into four groups (1) SPCF with TH < 2 mm and without syringomyelia (SPCF-TH0-only); (2) SPCF with the TH < 2 mm and with syringomyelia (SPCF-TH0-syr); (3) SPCF with the TH ≥ 5 mm without syringomyelia (SPCF-CM1-only), and (4) SPCF with the TH ≥ 5 mm with syringomyelia (SPCF-CM1-syr). We compared the four patient groups to an age and sex-matched control group. Compared to control, all four patient groups had significantly lower mid-sagittal PCF height, PCF area below the Twining line, and clivus and supraocciput length, consistent with hypoplasia of the inferior osseous PCF [17,20,22,24,27]. All four patient groups had a small, flattened (shallow), and overcrowded PCF compared to the control. The combined SPCF groups without syringomyelia compared to control demonstrated (1) reduced PCF height with reduced distance between neural and bony structures; (2) reduced distance between the cerebellar vermis and splenium of the corpus callosum consistent with a crowded PCF; (3) reduced volume of the cisterna magna, typical of classical CM1 and CM0 [24,70]; (4) retro-odontoid tissue hypertrophy constricting the ventral CSF space at the cervicomedullary junction; (5) shallowness of the PCF; (6) reduced CSF space within the FM [71]; and (7) caudal position of the obex and medulla [15]. The SPCF-TH0-only group, compared to the control, had reduced FM area and constriction of the ventral CSF space. The SPCF-TH0-only group but not the SPCF-CM1-only group had a narrowing of the ventral CSF pathway, reduced FM area, and reduced anteroposterior diameter of the spinal canal at the C1 level. The authors compared the SPCF-TH0-syr group to the SPCF-TH0-only group. They found that the SPCF-TH0-syr group had a significantly shorter anteroposterior diameter of the cervical canal at the first cervical vertebral (C1) level, more significant tapering of the cervical spinal canal, and greater PCF “crowdedness”, suggesting that upper cervical spinal canal narrowing and PCF crowding predispose to syringomyelia in CM0 patients [15,71]. In CM1 and syringomyelia patients, the spinal canal anteroposterior diameter is often narrower at C1 than at the FM [71]. The SPCF-TH0-syr group had a smaller anteroposterior spinal canal diameter at the C1 vertebra level than the SPCF-CM1-syr group [15]. The PCF was abnormally small in CM1 and SPCF-TH0 patients, and the hindbrain was abnormally caudal. In SPCF-TH0, boney hypoplasia, tightness, and neural crowding occurred immediately above and within the FM and the upper cervical spine. In contrast, in SPCF-CM1, hypoplasia reduced the PCF volume throughout the PCF. The SPCF-CM1-only group had more neural crowding in the superior part of the PCF than the SPCF-TH0 group. In patients with SPCF-TH0, narrowing of the inferior part of the PCF, the FM, and the upper cervical canal was associated with syringomyelia development [15]. Nishikawa et al. reported similar findings in patients with CM1 symptoms, SPCF, and the absence of significant TH [15].

Arachnoidal scarring in and around the FM may play a role in CM1 pathogenesis, CM0 symptomatology, and syringomyelia development [11,37,65,72,73]. Syringomyelia is significantly more likely in CM1 cases with arachnoid changes than without [65]. About half of operated CM0 and syringomyelia patients had arachnoid veils occluding the fourth ventricular outlets or arachnoid adhesions in the cisterna magna [11,37]. Arachnoid changes in CM1 spectrum disorders may be a primary or secondary phenomenon. FM arachnoiditis or tonsillar herniation can obstruct CSF flow through the cisterna magna. Kyoshima et al. described four adult patients without TH but with syringomyelia, tight cisterna magna, intraoperatively verified obstruction of foramen Magendie, and syrinx resolution after craniocervical decompression, consistent with CM0 [70]. Milhorat et al. reported obliteration of the cisterna magna in all their symptomatic CM1 patients [24].

Oldfield proposed that syringomyelia in CM1 arose from the cerebellar tonsils pulsating on an enclosed spinal subarachnoid space and creating enlarged CSF subarachnoid pressure waves that drive CSF into the spinal cord parenchyma [74,75,76]. The spinal subarachnoid space can be enclosed by lesser TH in the setting of SPCF, obliterated cisterna magna, and CM0, allowing tonsillar pulsation during the cardiac cycle to initiate syrinx formation and progression by the exact mechanism postulated in CM1. The cisterna magna is obliterated in about half of CM0 and CM1 patients and narrowed in the others [15]. Chiari-like symptoms may arise in patients with cisterna magna narrowing but without TH or syringomyelia. In patients with typical symptoms, narrowing of the cerebellar retrotonsillar CSF space supports a CM1 diagnosis [14,77]. Symptomatic CM1 patients have abnormal CSF flow and tonsillar pulsatility by imaging. In one study, phase-contrast cine-MRI of seven symptomatic adult patients with CM0-syr, suboccipital headache, neck pain, dizziness, and motor and sensory deficits demonstrated the severity of CSF circulation obstruction at the craniovertebral junction correlated with the severity of clinical symptomatology [69]. A crowded FM, cisterna magna arachnoidal adhesions, or a fourth ventricular outlet arachnoidal veil were surgically identified in the CM0 patients. Surgical decompression resolved CSF flow abnormalities and reduced syrinx size [69]. This study concluded that patients with CM0 clinically improved after CSF flow-restoring surgery, confirming that CM1-like pathophysiology could exist despite minimal or absent TH [69].

### 3.3. Risk of Bias in Studies: Epidemiology, Clinical Presentation, and Diagnosis of CM0

Using an MRI diagnostic threshold of 5 mm or more TH below the FM, a radiologic diagnosis of CM1 could be made in 0.8–0.9% of normal adults and 1.0–3.6% of children undergoing MRI in the US and the Netherlands [32,78,79,80]. The prevalence of symptomatic and asymptomatic CM0 is unknown. Available information in the literature is indirect and scarce. The estimated prevalence of symptomatic CM1 is 0.01–0.04%, although it could be substantially higher [18,78]. Surgical series of symptomatic patients have a higher percentage of CM1 patients (92–96%) than CM0 patients (4–8%) [37,39,81]. In a morphometric study of 333 patients with classical CM1 and 50 with CM0, the CM1 and CM0 cohorts had occipital bone hypoplasia of different types [20]. In another cohort of patients with CM1 signs and symptoms, 97 patients had MRI findings of CM0 (only 4% with the syrinx), 148 patients with MRI-borderline CM1, and 183 with MRI-verified CM1 with TH > 5 mm [16]. The prevalence on MRI of SPCF, short clivus length, and tight cisterna magna in the US is 8% [82]. Therefore, that population could harbor occult, latent asymptomatic, and minimally symptomatic CM0.

There is a weak relationship between the extent of TH and the severity of CM1 symptoms [16]. Therefore, the amount of tonsillar ectopia on MRI cannot serve as the sole diagnostic criterion for validating or refuting that an individual’s symptoms arise from CM1 or its variants. In CM1, FM and PCF crowding measures may be better than TH extent in predicting which patients have CM1 symptoms [15,20,40,56,68,83,84]. Patients with CM0-only display symptoms and signs like CM1 and can be categorized by (1) FM obstruction, (2) brainstem caudal displacement and brainstem or cerebellar compression, (3) traction on cranial nerves, and (4) central myelopathy from syringomyelia [15,16,22,27,35,37,42]. Patients with SPCF without TH and other CM1 spectrum subtypes continue to experience delays in diagnosis and misdiagnoses. The average period from the first physician visit to a CM1 diagnosis in the US was reported as 3.4 years [85].

Headaches and neck pain are the most common complaints of CM1 and CM0 patients [15,16,24,30,85,86]. The cerebellar tonsils applying pressure to the surrounding dura or obstructing CSF flow at the FM probably provokes this headache. Pathognomonic occipital headaches are more common in presurgical patients than non-surgical patients [24,30,87]. The prevalence of headaches and 28 other clinical symptoms did not differ between three groups of symptomatic CM1 spectrum patients with “zero” (0–2 mm), borderline (3–5 mm), and full TH (>5 mm) below the FM [16]. Most CM0 and CM1 patients report otoneurological disturbances, including dizziness, vertigo, disequilibrium, and decreased hearing [24,85]. These symptoms were verified by an otoneurological examination in patients with less than 5 mm tonsillar ectopia, confirming their occurrence in CM0 patients. Mild or nonspecific symptoms of non-Valsalva-related generalized headache and neck pain, tinnitus, numbness, fatigue, memory impairment, and “brain fog” are common in patients with SPCF and CM0 or CM1. Because these symptoms are nonspecific to CM0 and CM1, reliable MRI diagnostic measures should be used to evaluate SPCF in those patients. In one study, six MRI morphometric measures of the PCF were compared between healthy persons with normal (<0 mm) and borderline TH (1–5 mm) and symptomatic patients with borderline (1–5 mm) and severe (6–13 mm, CM1) TH [21]. A comparison of the two control groups of asymptomatic subjects with normal (<0 mm) and low-lying tonsils (1–5 mm) showed that PCF area was significantly less in those with low-lying tonsils, suggesting that subclinical SPCF caused the low-lying cerebellar tonsils (and low fastigium) in that group [21]. Symptomatic CM0 and borderline CM1 patients significantly differed from the healthy controls without tonsillar ectopia on four measurements. The group of symptomatic patients with borderline TH (1–5 mm) had a shorter clivus than asymptomatic patients with borderline TH but no other significant measurement differences. The finding that only symptomatic patients with low-lying tonsils had a short clivus suggested that finding a short clivus in patients with atypical symptoms could validate that those symptoms were related to low-lying tonsils. Symptomatic CM1 patients with severe TH had significantly different PCF measurements compared to the healthy controls [15].

Ventrolateral TH (the tonsils extending anterior to a line bisecting the caudal medulla at the FM level on axial MRI) is a relatively common radiographic finding in adult CM0 and CM1 patients [68]. Ventrolateral TH can compress the lateral brainstem nuclei and cranial nerves, unlike caudal TH, which compresses the dorsal brainstem and obliterates the CSF space between the dorsal brainstem and tonsils [1,68]. Ventrolateral TH (mm) is independent of the extent (mm) of caudal TH on sagittal MRI. Ventrolateral TH can produce Chiari-like symptoms in patients without TH below the FM. Headache is significantly more likely in adult patients with than without ventrolateral TH [68]. Medullary symptoms in young children with ventrolateral TH and less than 5 mm of TH improved clinically with PCF decompression [1]. Ventrolateral tonsillar ectopy is, therefore, a clinically relevant radiographic measure to evaluate, especially in patients with Chiari-like symptoms but without TH on midsagittal MRI [68].

### 3.4. Synthesis: Using Automated Radiographic Measurements May Reduce the Risk of Bias in Studies of CM0 

Automated radiographic measurement of PCF volume, dorsal TH, ventrolateral TH, and basilar invagination (BI) could improve the detection of CM0 and CM1. Post-processing diagnostic methods driven by machine learning may lead to better detection of CM0 [40,88]. MRI or CT morphometric analysis can also identify CM0 with focal hypoplasia restricted to the inferior PCF and FM region [1,16,20,39,68,69,71,89,90].

## 4. Discussion

Craniocervical decompression is indicated in CM1 and CM0 patients with brainstem dysfunction, cranial nerve deficit, symptomatic syringomyelia, and life-dominating occipital tussive headache [35,67]. Most patients with severe symptoms and clinical signs undergo surgical management. In patients with nonspecific or less severe symptomatology, imaging features of boney hypoplasia of the PCF, FM, and upper cervical canal would support a diagnosis of symptomatic CM0. Latent CM0 and SPCF predispose to the development of acute FM syndrome from lumbar puncture, bilateral trigeminal neuralgia, primary cough headache, and the need for surgical decompression to treat PCF hemorrhage [91,92,93,94]. Morphometric analysis of the PCF can diagnose unsuspected CM0 and SPCF.

Syrinx morphology and spinal location differ according to syrinx etiology [95]. Generally, syringes associated with CM1 and CM0 extended more superiorly in the cervical spine than syringes of spinal origin [27,95]. The width of syringes in adult patients with CM0 is similar to or narrower than in patients with CM1 [27,95]. Pediatric patients with CM0 presented with syringes of various lengths [37]. The similarity in syrinx morphology between CM1 and CM0 patients is consistent with shared syrinx pathophysiology.

This study has several limitations. The number of studies was limited, as CM0 cases defined by the original criteria, including syringomyelia, are uncommon. Studies reporting CM0 cases without syringomyelia only appeared in the past few years. The sample sizes in several studies were small. The various studies did not use the same definitions. Most studies consisted of surgical series. The studies differed in their patient demographics. Usually, symptomatic patients were compared to normal control subjects rather than asymptomatic patients with minimal tonsillar ectopia. Our review considers the limitations of previous studies in reporting current thinking about the place of CM0 within Chiari 1 spectrum malformations.

## 5. Conclusions

Classical CM1 and CM0 may occupy the same phenotypical continuum and have multifactorial etiologies. MRI morphometric characteristics of CM1 and CM0 include isolated congenital occipital bone hypoplasia and alterations of other skull base bones affecting PCF configuration and volume. The CM0 subtype also has absent or minimal TH on midsagittal MRI, a shallow boney “funnel” around the FM, narrow CSF spaces, and squeezing of neural elements on axial MRI (Figure 2). The CM0 subtype, like CM1, may be associated with syringomyelia, presumably resulting from disturbed CSF flow at the foramen magnum. Specific CM1-spectrum symptoms and signs are required to diagnose CM0 without syringomyelia because MRI morphometric abnormalities typical of CM0 can be seen in asymptomatic patients or those with other conditions. Because the natural history of patients with minimally symptomatic CM0, radiologically identified SPCF, and absence of syringomyelia is unclear, more research is needed to study the morphological and clinical findings of these patients and their potential implications for surgical decision-making. The benefit of surgical treatment in CM0 without syringomyelia should be evaluated by prospective, controlled trials comparing the outcomes of surgical and non-surgical treatments.

## Data Availability

Not applicable.
